# Medical Defence Union and the London and Counties Medical Protection Society (Limited)

**Published:** 1893-12-30

**Authors:** Leslie Phillips, A. George Bateman

**Affiliations:** 20, Craven Street, Charing Cross, London, W.C.; 20, Craven Street, Charing Cross, London, W.C.


### Editor's Letter-Box.

[Our correspondents are reminded that prolixity is a great bar to publication, and that brevity of style and conciseness of statement greatly facilitate early insertion.]

### MEDICAL DEFENCE UNION AND THE LONDON AND COUNTIES MEDICAL PROTECTION SOCIETY (LIMITED).

In your issue of the 9th there appears a letter signed, we regret to see, with the names of Mr. Bruce Clarke and Dr. Heron. In this letter certain grave charges are made against the Council of the Defence Union relative to the facts enumerated in their *résumé* of the recent negotiations.

Charge No. 1.—That in the 29th paragraph of the *résumé* the statement that the London and Counties Protection Society contemplate winding up is untrue.

In answer to this charge we have only to say that if Messrs. Clarke and Heron choose to tear out from a sentence in the *résumé* a few words, and try to lead your readers into the belief that the Council of the Defence Union made the above statement without context or qualification, such conduct will only bring with it its own condemnation. In common fairness and honesty of purpose Messrs. Clarke and Heron ought to have quoted the whole paragraph, as then your readers would have seen that it was but one of a large number all depending on each other, and that the accusation consequently is insincere.

Messrs. Clarke and Heron also, although compelled to admit that the subject was discussed, neglect to mention that the point was finally settled during the negotiations, and that the proposal that the London and Counties Protection Society should wind up was determined upon, not only on the agreement of the negotiators, but also on the decisive opinion of both solicitors. It appears to us that by these omissions it is Messrs. Clarke and Heron who have committed suppression of material fact, and we repeat that the paragraph in the *résumé*, if it be honestly read and not multilated, is an absolutely accurate record of the negotiations.

Charge No. 2.—That the Council of the Defence Union suppressed a "counter-suggestion" of the Protection Society. This charge was originally made in what Mresss. Clarke and Heron, with seemingly involuntary truth, allude to as the "hasty reply" of Dr. Woods "without consultation with his other two colleagues." In our refutation of Dr. Woods' "hasty" mis-statement of facts, and published in your issue of the 9th, your readers will find that the meetings were brought to an end by Dr. Heron, who declared that if the Council of the Defence Union adhered to their last proposal (and here we use his own words) "the negotiations were at an end." As we have already stated, the so-called "counter-suggestion" was utterly meaningless, and, properly treated as such by our Council, for it had already been agreed to leave the Executive of the Defence Union as it stood, and to admit the twenty nominees of the Protection Society as Vice-Presidents. The Council of the Defence Union therefore suppressed nothing in their *résumé*, for they ended their history of the negotiations where these latter had been terminated by the action of the Protection Society's representatives.

Charge No. 3.—That not till November 28th had they any opportunity "of knowing the fate of their 'counter-suggestion.'" This statement is the most regrettable of all, for it compels us to make public the fact that, not only had the above points been fully made clearly to Messrs. Clarke and Heron long before the 28th instant, but also that these two gentlemen actually called on the President of the Union (Mr. Horsley) as early as November 22nd to learn from him what would be the probable result of the Council's action at the usual fortnightly meeting to be held that afternoon. Messrs. Clarke and Heron were thereupon informed that there was no probability that the Council would alter its original proposition, and which was rejected by the Protection Society. Messrs. Clarke and Heron then left, repeating their former statement (*vide supra*), that if that were so then the negotiations were at an end. To say, therefore, as they do now, that they "had no opportunity" of knowing how matters stood till a week later, namely, November 28th, is already not true in fact. From certain hints and indications in the letter it would seem to appear that Messrs. Clarke, Heron, and Woods wish the profession to understand that the negotiations have all along been unreal on their part, and that while the Council of the Defence Union imagined it was negotiating with the Protection Society, as arranged by the two societies on July 26th, 1893 (*vide* the *résumé*, paragraphs 4 *et seq.*), it was, as a matter of fact, dealing unconsciously with an oligarchy of only three, or more correctly, as Dr. Woods rarely attended, but two persons totally devoid of any responsibility.

If such a state of things is seriously meant by these gentlemen to be understood to have existed, then we must confess it would appear to us to be not merely a discredit to any body of men dealing with a plain business matter, but also a betrayal of the best interests of the whole profession as well as those of the members of the Protection Society.—We remain, yours faithfully,

Leslie Phillips, M.D.
A. George Bateman, M.B.

P.S.—We append a letter received from the solicitor, Mr. Higham, who attended the meeting of the committee as stated in the *résumé* on behalf of the Defence Union.

20, Craven Street, Charing Cross, London, W.C., December 12th, 1893.

Dear Mr. Bateman.—My recollection of what took place at the meeting between the representatives of the Medical Defence Union and of the London and Counties Medical Protection Society on the 24th of October last is as follows: The solicitors on each side were asked to advise whether the two companies could be bodily amalgamated, and we replied that there was no power in the articles of association of either institution to effect this; that the only course would be for the smaller society to go into liquidation, and for its members then to join individually the Medical Defence Union. In answer to further questions, we advised that the rights and privi eges conferred on its members by the M. D. U. were equivalent to those offered by the L. and C. M. D. S., and that the costs of the liquidation of the latter body could not legally be paid out of the funds of the Defence Union. We also replied to several incidental points raised by those who were present as to the mode in which the liquidator would have to deal with the assets and liabilities of the L. and C. M. Protection Society. I certainly understood that liquidation was not only contemplated by the Sub-Committee of the L. and C. M. Protection Society, but that they and their solicitor acquiesced in this as the only fea ible method of amalgamation.—Yours faithfully,

A. C. Bateman, Esq. M.D. S. Stagoll Higham.

